# Multiomics Picture of Obesity in Young Adults

**DOI:** 10.3390/biology13040272

**Published:** 2024-04-18

**Authors:** Olga I. Kiseleva, Mikhail A. Pyatnitskiy, Viktoriia A. Arzumanian, Ilya Y. Kurbatov, Valery V. Ilinsky, Ekaterina V. Ilgisonis, Oksana A. Plotnikova, Khaider K. Sharafetdinov, Victor A. Tutelyan, Dmitry B. Nikityuk, Elena A. Ponomarenko, Ekaterina V. Poverennaya

**Affiliations:** 1 Institute of Biomedical Chemistry, Moscow 119121, Russia; olly.kiseleva@gmail.com (O.I.K.);; 2 Faculty of Computer Science, National Research University Higher School of Economics, Moscow 101000, Russia; 3 Research Department, Eligens S.I.A., LV-2167 Marupes, Latvia; 4 Federal Research Centre of Nutrition, Biotechnology and Food Safety, Russian Academy of Sciences, Moscow 109240, Russia; 5 Russian Medical Academy of Continuing Professional Education, Ministry of Health of the Russian Federation, Moscow 125993, Russia; 6 I.M. Sechenov First Moscow State Medical University (Sechenov University), Ministry of Health of the Russian Federation, Moscow 119991, Russia

**Keywords:** obesity, young adults, BMI, genomics, proteomics, metabolomics, multiomics, predictive models

## Abstract

**Simple Summary:**

Obesity is a significant health concern associated with fat accumulation and complications like inflammation, cancer, and diabetes. Understanding its molecular roots is crucial, especially for young individuals who are capable of lifestyle changes. Our study examined underweight, lean, overweight, and obese individuals by analyzing blood samples using metabolomics, proteomics, and genomics. Using metabolomics, we identified 313 substances; proteomics revealed 708 proteins, and genomics explored 647,250 point mutations. Models predicting body mass index showed that, individually, proteomics provided more value for prediction, followed by metabolomics and genomics. Combining proteomics and metabolomics in a multiomic approach yielded the best results, surpassing single-factor analyses. This pioneering study is the first to focus on obesity in young people, integrating genomic, proteomic, and metabolomic data. Our findings provide valuable insights into the molecular mechanisms of obesity, offering potential for more targeted interventions.

**Abstract:**

Obesity is a socially significant disease that is characterized by a disproportionate accumulation of fat. It is also associated with chronic inflammation, cancer, diabetes, and other comorbidities. Investigating biomarkers and pathological processes linked to obesity is especially vital for young individuals, given their increased potential for lifestyle modifications. By comparing the genetic, proteomic, and metabolomic profiles of individuals categorized as underweight, normal, overweight, and obese, we aimed to determine which omics layer most accurately reflects the phenotypic changes in an organism that result from obesity. We profiled blood plasma samples by employing three omics methodologies. The untargeted GC×GC–MS metabolomics approach identified 313 metabolites. To augment the metabolomic dataset, we integrated a label-free HPLC–MS/MS proteomics method, leading to the identification of 708 proteins. The genomic layer encompassed the genotyping of 647,250 SNPs. Utilizing omics data, we trained sparse Partial Least Squares models to predict body mass index. Molecular features exhibiting frequently non-zero coefficients were selected as potential biomarkers, and we further explored enriched biological pathways. Proteomics was the most effective in single-omics analyses, with a median absolute error (MAE) of 5.44 ± 0.31 kg/m^2^, incorporating an average of 24 proteins per model. Metabolomics showed slightly lower performance (MAE = 6.06 ± 0.33 kg/m^2^), followed by genomics (MAE = 6.20 ± 0.34 kg/m^2^). As expected, multiomic models demonstrated better accuracy, particularly the combination of proteomics and metabolomics (MAE = 4.77 ± 0.33 kg/m^2^), while including genomics data did not enhance the results. This manuscript is the first multiomics study of obesity in a gender-balanced cohort of young adults profiled by genomic, proteomic, and metabolomic methods. The comprehensive approach provides novel insights into the molecular mechanisms of obesity, opening avenues for more targeted interventions.

## 1. Introduction

Obesity, defined by a body mass index (BMI) equal to or exceeding 30.0 kg/m^2^ [1], has tripled over the past three decades and continues to proliferate across developed and developing nations. According to projections from the World Health Organization, it is anticipated that one in every five adults globally will suffer from obesity by 2025. Despite the escalating recognition of the multifaceted nature of this disease, scientific focus on obesity remains disproportionately inadequate compared to its impact [2].

With the increase in the number of overweight individuals [3], the very concept of “obesity” has transformed. Whereas earlier this term indicated the accumulation of adipose tissue, one of the contemporary hallmarks of obesity is chronic inflammation [4].

Causative pathologies contributing to obesity encompass hypothyroidism [5], Cushing syndrome [6], polycystic ovarian syndrome [7], hypogonadism [8], and growth hormone deficiency [9]. Contributing factors include medication (psychiatric drugs, corticosteroids, insulin, and specific β-adrenergic receptor blockers), mental factors (chronic stress, depression), lifestyle changes (crash diets, smoking cessation), sleeping disorders, and alcoholism [10]. Epidemiological studies highlight obesity’s strong contributions to cancer, cardiovascular diseases, type 2 diabetes, liver diseases, and other disorders [11].

Of particular concern is the prevalence of obesity among youth [12,13]. The cohort of young adults manifests considerably higher BMIs at an earlier life stage than preceding generations [14]. Beyond the evident medical costs, adolescence-onset obesity can engender social stigma and decrease life expectancy.

Given the complexity of obesity as a multifaceted disease influenced by genetic, environmental, and lifestyle factors, employing omics techniques is essential to investigate the underlying intricate molecular mechanisms comprehensively. These methods enable a holistic understanding by concurrently examining genomic, transcriptomic, proteomic, metabolomic, and epigenomic aspects and have the potential to reveal potential targets for more effective interventions.

Over the last two decades, many research projects have been carried out to identify molecular changes associated with obesity. Attempts have been undertaken to pinpoint genetic variations in individuals with obesity through GWAS (genome-wide association studies) within extensive sample collections encompassing hundreds of thousands of patients [15]. For example, one GWAS identified over 97 loci associated with BMI [16] and 49 loci linked to waist-to-hip ratio [17]. Additionally, a more extensive meta-analysis involving 700,000 individuals revealed a connection between over 941 single-nucleotide polymorphisms and BMI [18]. In early GWAS, a cluster of loci within the intron of FTO was identified, exhibiting a relatively substantial impact on BMI (0.35 kg/m^2^ per allele) [19].

Nevertheless, the risk associated with most analyzed loci proved statistically insignificant. At the genomic level, the annotation of rare, early-onset, and severe monogenic obesity was also conducted. This form of obesity follows a Mendelian inheritance pattern and is linked to variants in a limited number of genes, such as MC4R, POMC, LEP, and LEPR [20].

Of particular note is the pan-European DioGENES project, within which a longitudinal proteomic analysis of a large cohort of overweight and obese individuals with weight (re)gain was performed [21,22]. Proteomic profiling of 400+ patients identified 39 proteins associated with obesity and weight loss, including not only proteins previously studied in the context of obesity (in particular, adiponectin, C-reactive protein, and calprotectin), but also new putative biomarkers for weight loss/maintenance (e.g., D109 antigen and PRAP1).

In metabolomics, the cohort of six metabolic pathways, whose oscillations facilitate the predictive power of obesity risk, were estimated. These pathways encompass the metabolism of glucose, choline, creatine, fatty, bile, and amino acids, as well as the citric acid cycle [15]. Subsequent scrutiny of metabolic pathways involving fatty acid biosynthesis, phenylalanine metabolism, and leucine/valine degradation holds promise for elucidating distinctions between metabolically healthy and unhealthy obese individuals [23].

Integrating multiomic data can help detect specific biomarkers in people with a risk profile and take steps to normalize the condition before obesity develops [15]. Integrating omics data is imperative in formulating precision strategies for obesity prevention [2,15] and switching from the one-size-fits-all approach to an individualized-care solution. Thus, through the integration of epigenomic, genomic, transcriptomic, and metabolomic data, researchers have been able to unravel cross-connections between biological molecules and networks that are fundamental to the development of obesity and other complex phenotypes [15,24]. An example from the scarce body of publications integrating metabolomic, proteomic, and genomic levels is a recent comprehensive study examining the interplay between multiomic signatures and BMI. The study presented an atlas of changes in 1111 blood analytes associated with BMI alterations, along with multiomic associations with polygenic risk scores and the composition of the gut microbiome in a cohort of 1277 individuals with a mean age of 44 ± 10 years [14].

Consequently, our investigation into obesity has persisted, with a pronounced emphasis on multiomics. This work is a logical extension of the research [25] conducted at the proteomic-only level. We demonstrated the impossibility of categorizing patients based solely on the results of standard blood tests; however, the identified proteomic patterns provide additional information about the patient’s phenotype for more personalized treatment. In this study, we expanded the area of molecular interest into genomics and metabolomics.

It is pertinent to acknowledge that we are not the first to conduct multiomic analyses on individuals with obesity. The pioneering work of Zhang et al. in 2022 aimed to simultaneously detect the causal relationship among multiomics obesity-associated data sets from gene expression, methylation, and metabolites [24]. The initial goals of our works are related, but there are several significant differences. As noted, we analyzed different omics levels (genomics, epigenomics, transcriptomics, and metabolomics in [24] vs. genomics, proteomics, and metabolomics in our work). Moreover, it is essential to note that, despite the similar size of the patient samples (104 vs. 101), the population of these samples is significantly different. Thus, the article by Zhang et al. [24] is based only on the molecular profiles of young women with different bone mineral densities (obtained as part of a large-scale project to study osteoporosis), which may not be generalizable to males. Our study analyzed several omics layers of males and females (50:51) with different body mass indices, which were collected based on their weight at the time of their hospitalization in a nutrition clinic without reference to any other pathologies except for obesity. It is also essential to highlight the differences in methods for statistical interpretation of results. In Zhang et al. [24], the emphasis was on Mendelian randomization analysis for a holistic understanding of molecular regulatory information flow in obesity. We, in turn, utilized single- and combined-omics data, trained sparse Partial Least Squares models to predict body mass index, and selected molecular features that exhibited frequently non-zero coefficients as potential biomarkers in parallel.

To the best of our knowledge, this is the first effort of its kind made on a gender-balanced cohort of young overweight and obese adults compared with healthy lean (and slightly underweight) volunteers.

## 2. Materials and Methods

### 2.1. Subjects

A total of 101 fasted human plasma samples were obtained from the patients of the Clinic of “The Federal Research Center of Nutrition and Biotechnology” (Moscow, Russia).

The inclusion criteria were the study participants’ ages of 18 to 45. Individuals with diagnosed somatic and mental disorders (other than obesity), pregnant/breastfeeding patients, and patients who underwent bariatric surgery or experienced recent (<6 months) weight loss were excluded from the study.

Anthropometric examinations were conducted for all participants, including body weight and height measurements, to calculate BMI [body weight [kg]/(body height [m])^2^]. The participant cohort exclusively consisted of individuals of Caucasian ethnicity. All patients were aged beyond 44 years to align with the World Health Organization’s delineation of young adulthood [26]. All study participants provided informed consent, confirming their willingness to participate in the research. The ethics committee of the Institute of Biomedical Chemistry approved the study.

### 2.2. Genomic Analysis

Genomic DNA was extracted using the QIAamp D.N.A. Blood Mini Kit (Qiagen, Hilden, Germany) according to the manufacturer’s protocol. This kit is designed for efficient isolation of high-quality DNA from blood samples. The quality of the extracted DNA was assessed using agarose gel electrophoresis. Electrophoresis in agarose gel was conducted to confirm the DNA samples’ integrity and purity. DNA concentration was estimated using a Qubit 3.0 fluorometer (Thermo Fisher Scientific, Waltham, MA, USA). The sample preparation protocol for genotyping and CNV analysis (Infinium HTS Assay, Illumina, San Diego, CA, USA) does not require additional sample quality assessment using a spectrophotometer.

The genetics data were initially obtained for only 96 samples due to the nature of sample batch processing. Two samples were further excluded from the initial sample collection because they were obtained from patients aged 45 years old and did not satisfy the World Health Organization’s delineation of young adulthood. All samples were found to be suitable for subsequent analyses. Sample preparation and chip scanning were performed on the HiScan system (Illumina, San Diego, CA, USA) following the Infinium HTS Assay Guide. Infinium Global Screening Array-24 v. 2.0 chips were employed for the analysis. Genotyping of the samples was carried out using Illumina AutoConvert Software v. 2.0.1, with the AutosomalCallRateThreshold parameter set to 0.01. This threshold was chosen to ensure high data quality in the genotyping process. Following genotyping, an internal quality control step was implemented to filter out low-quality genotype calls, ensuring the reliability of the obtained genotypic data for further analysis.

### 2.3. Proteomic Analysis

The samples were employed for standard proteomic profiling. The detailed methodology is presented in the article by Kiseleva et al. [25]. Briefly, to enhance the detection of lower abundance proteins, we depleted high-abundance proteins such as albumin and IgG from blood plasma using the ProteoPrep Kit (Sigma-Aldrich, Burlington, VT, USA). This was followed by trypsinolysis of the depleted plasma and subsequent analysis using an Ultimate 3000 nano-flow HPLC system connected to an Orbitrap Exactive mass spectrometer (Thermo Fisher Scientific, Waltham, MA, USA).

To process proteome data, we used SearchGUI software [27] (v. 4.1.24, search engines were X!Tandem, MS-GF+, OMMSA) against SwissProt library of human canonical and alternatively spliced protein sequences in automatic mode [28]. The parameters remained consistent with those outlined in the previous proteomic article [25]. We used the normalized spectral abundance factor (NSAF) for label-free quantification proteomics [29]. This detail sets the current study apart from our previous research [25], where protein abundance was treated as a binary variable.

The MS data were deposited to the ProteomeXchange Consortium via the PRIDE partner repository [30] with the dataset identifier PXD023526. Quality control analysis is presented in Supplementary Document S1 and was deposited to Mendeley Data (dataset identifier 10.17632/t255cjz787.1).

### 2.4. Metabolomic Analysis

#### 2.4.1. Sample Preparation for Metabolomic Analysis

Sample preparation was conducted to analyze blood plasma’s low-molecular-weight fraction, including quenching, extraction, methoximation, and derivatization [31,32]. The low-molecular-weight fraction of the blood plasma sample (30 µL) was sequentially extracted with a mixture of isopropanol, acetonitrile, and water (3:3:2, *v*/*v*/*v*), then further extracted with a mixture of acetonitrile and water (1:1, *v*/*v*); samples were centrifuged after each extraction. Next, the supernatant was evaporated to dryness in a SpeedVac evaporator (Concentrator plus, Eppendorf, Hamburg, Germany) and oxidized with 10 µL of freshly prepared methoxyamine hydrochloride (20 mg/mL in pyridine) at 30 °C for 90 min on a thermoshaker (ThermoMixer C, Eppendorf, Hamburg, Germany) at 1300 rpm. The samples were derivatized by 91 µL of N-methyl-N-(trimethylsilyl)-trifluoroacetamide (MSTFA) with a mixture of fatty acid methyl esters (FAMEs) at 37 °C for 30 min in a thermoshaker at 1300 rpm. After extraction and derivatization, samples were submitted for GC×GC–MS acquisition [32].

#### 2.4.2. GC×GC–MS Analysis

An untargeted metabolomics approach was used to detect metabolites. GC×GC–MS analysis was carried out on a 7890B chromatography system (Agilent Technologies, Santa Clara, CA, USA) and a time-of-flight mass spectrometer Pegasus BT 4D (LECO, St. Joseph, MI, USA) equipped with an L-PAL3 autosampler (PAL Systems, Zwingen, Switzerland). Each prepared sample (1 µL) was injected through the glass liner (Restek, Centre County, PA, USA) under split mode (50:1). Helium (6.0 grade) was used as a carrier gas, and a constant flow of 1 mL/min was maintained throughout the run. The oven was initially heated to 60 °C, the equilibration time was 1 min, and the temperature was ramped at a 10 °C/min rate to the final temperature of 280 °C, with a hold time of 12 min. The first-dimension column was 30 m long Rxi-5Sil MS (Restek, Centre County, PA, USA), and the second-dimension column was 3 m long Rxi-17Sil MS (Restek, Centre County, PA, USA). The transfer line of the time of MS was set at 280 °C, with a solvent delay of 350 s. The ion source temperature was 250 °C. Spectra were collected from 35 to 700 *m*/*z* at 70 eV electron ionization energy. The scan rate was 200 spectra per second. Data were acquired by ChromaTOF software (v. 5.51, LECO, St. Joseph, MI, USA). Each sample was analyzed in at least three repetitions to minimize the technical error. To eliminate the possibility of cross-contamination between samples and to monitor signals unrelated to biological extracts, a method blank sample and pure pyridine were injected every three GC×GC–MS runs. Quality control also included checking reproducibility by monitoring retention times and areas under the chromatographic curves of FAME added to extracts and blank samples. The coefficient of variations of areas under chromatographic peaks for triplicate injections for monitored FAMEs did not exceed 15% (on average, <6%).

Raw GC×GC–MS data were submitted to the MetaboLights repository, have a permanent unique identifier MTBLS8961, and can be found at www.ebi.ac.uk/metabolights/MTBLS8961 (accessed on 25 December 2023). The dataset is also available from the corresponding author upon request. Quality control of the metabolomics dataset is provided in Supplementary Document S1.

### 2.5. Data Processing and Statistical Analysis

Data preprocessing included log-transform, normalization to zero mean, and unit variance. Adjustment for known batches was carried out via an empirical Bayesian framework using ComBat software implemented in the SVA library v. 3.50.0 [33]. 

Sparse Partial Least Square (sPLS) regression was used to predict patient BMI based on omics data. We utilized the sPLS implementation in the mixOmics package v. 6.0 [34]. The 10-fold cross-validation procedure was implemented to mitigate the risk of overfitting and provide a robust evaluation of the model’s performance. A sparse PLS model with one latent component was trained for each cross-validation run, with BMI as a dependent variable and omics data as predictors. As the best practices described in the mixOmics manual suggested, the optimal number of features for a latent component was determined using 50 rounds of a 10-fold cross-validation cycle. This second level of cross-validation to optimize the number of significant features did not include samples from the external cross-validation cycle that were used to estimate overall model performance.

Omics features selected by the sPLS model as significant BMI predictors were subjected to overrepresentation analysis using the ClusterProfiler package [35]. Pathway composition data was downloaded from Wikipathways [36] and the Human Metabolome database [37]. All *p*-values resulting from enrichment analysis were corrected for multiple testing using the FDR approach [38].

## 3. Results

A total of 101 plasma samples were obtained from the obese patients and healthy volunteers. All patients were classified as young (no older than 44 years old) with a BMI range from 17.4 to 62.5 kg/m^2^, while the mean BMI was 32.8 ± 9.2 kg/m^2^. The male-to-female ratio was 50:51. The age range was from 18 to 44 years, with mean age equal to 31.6 ± 6.7 years (Supplementary Table S1). Descriptive statistics of patient characteristics enrolled in the study are presented in Table 1.

**Figure 1:**
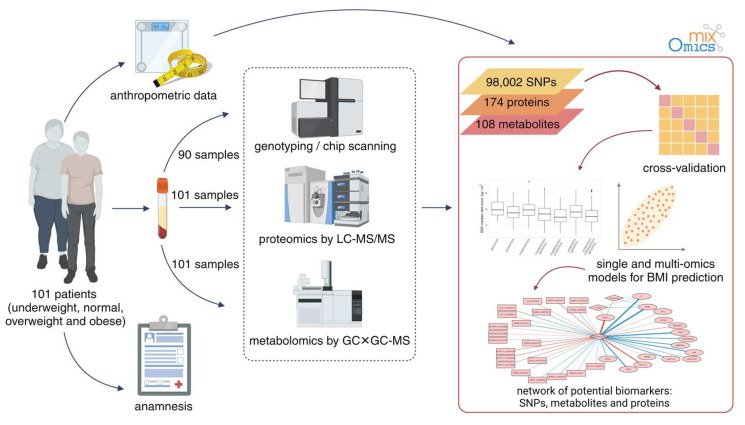
The overall workflow of the experiment to investigate the molecular mechanisms of obesity.

The experiment setup is illustrated in Figure 1. Briefly, samples were characterized using a multiomic approach. This included proteomic (semiquantitative HPLC–MS/MS) and metabolomic (GC×GC–MS) profiling of sample blood for 101 patients, and the genotyping data (647,250 SNPs, Illumina Infinium Global Screening Array-24 v2.0 chips) were available for 90 out of 101 patients.

To investigate relationships between omics data and obesity, we used sparse Partial Least Square (sPLS) regression. This data analysis technique provides dimensionality reduction and feature selection, effectively handling high-dimensional data while improving model interpretability and predictive performance. The sparsity in sPLS models is beneficial for omics data because it reduces overfitting, enhances interpretability, and narrows the focus to a subset of analytes that have the most significant impact on the biological processes of interest. We used sPLS implementation from the mixOmics package [34]. 

**Figure 2:**
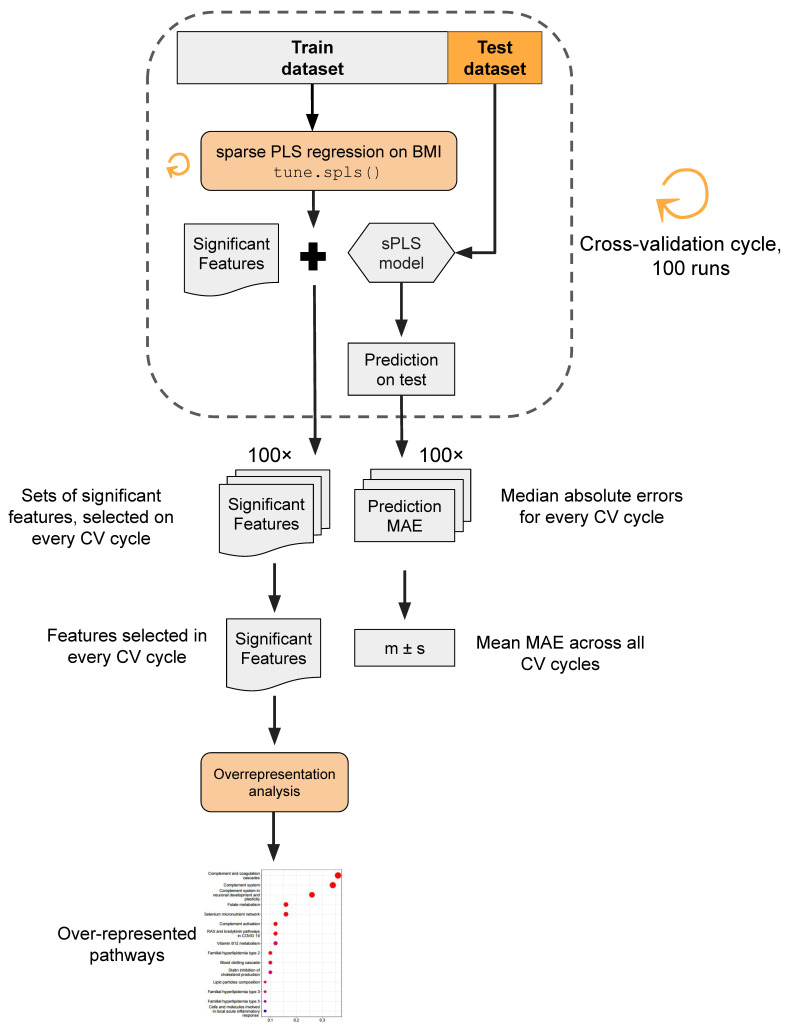
Data analysis workflow. In brief, sPLS regression was used to predict patient BMI based on three distinct omics layers. The cross-validation procedure was implemented to mitigate the risk of overfitting. The model’s performance was assessed via the median absolute error metric (MAE). Features selected as significant BMI predictors were subjected to overrepresentation analysis.

The analytical pipeline proceeded as follows (Figure 2). Utilizing sPLS regression, we constructed sparse models to predict patient BMI based on each of the three distinct omics layers. Given the supervised nature of sPLS, a cross-validation approach was employed to prevent overly optimistic predictions. In each iteration of the cross-validation cycle (in total, executed 100 times), 10 random samples were selected as a test dataset, while the remaining 91 samples constituted the training dataset. The BMI prediction model employing the tune.spls() function was trained, and its performance was assessed on the test dataset. Within every cycle of cross-validation, the model training yielded a set of omics features selected by sPLS as significant, signifying non-zero coefficients in the model. Model performance was estimated using the median absolute error (MAE) metric, representing the median difference between the true BMI values and model predictions. Molecular entities identified as significant features across cross-validation iterations underwent overrepresentation analysis to unveil pathways potentially linked to obesity development in young adults.

Our analysis was intentionally limited to samples from young adults (18–44 years old). It is generally reported that BMI depends on age and gender in the overall population [40]. However, according to the linear modeling results, age and gender were non-significant BMI predictors in our patient cohort. Hence, age and gender were not included as covariates in the sparse omics-based models. In the following sections, we describe the results of each omics data analysis.

### 3.1. Genome Analysis

Genotyping was used to build models for BMI prediction through the cross-validation procedure. In contrast to proteomics and metabolomics profiling, genotyping data were obtained for 96 patients who were genotyped for 647,250 SNPs using Illumina Infinium Global Screening Array-24 v2.0 chips due to sample batch processing. Two samples were excluded from the study because they were obtained from patients aged 45 y.o. and hence did not satisfy the World Health Organization’s delineation of young adulthood. Two samples were found to be duplicates, and two samples had a call rate of less than 0.9. Thus, the genotyping data were available for a total of 90 patients. 

Extensive SNP filtering was performed to narrow down the feature space. We focused on SNPs that could be unambiguously mapped to the protein-coding genes on the autosome chromosomes. We also removed SNPs with unknown genotypes in three or more patients. Finally, we filtered out SNPs with low variance according to the nearZeroVar() function from the mixOmics package [34].

After filtering, 98,002 variants were used to train 100 sparse PLS models for BMI prediction. The average prediction accuracy across all models was 6.14 ± 0.37 kg/m^2^ (mean ± 95% confidence interval). For the interpretation at the gene level, we mapped the similarity to proteomics and metabolomics analyses; for each SNP, we calculated how frequently (i.e., in how many cycles of cross-validation) it had a non-zero coefficient in the BMI prediction model. 

Gene-level results are presented in Supplementary Table S2. Here, we discuss several genes associated with glucose homeostasis, lipid metabolism, serotonin bioavailability, and phospholipid transport, with many SNPs consistently found to have non-zero coefficients for BMI prediction models. 

Gene PDK3 (encoding pyruvate dehydrogenase kinase 3, Q15120) plays a role in glucose homeostasis and maintaining normal blood glucose levels. It has been shown that the expression of PDK3 increases in obese individuals [41]. Gene PCYT1B (phosphate cytidylyltransferase 1B, choline, Q9Y5K3) is involved in lipid metabolism, potentially linking it to obesity or metabolic disorders. SLC6A4 (solute carrier family 6 member 4, P31645) is an essential mediator of serotonin bioavailability and is crucial in regulating energy balance and appetite control. The methylation analysis of this gene showed that people with obesity had lower methylation of SLC6A4 CpG5 and lower expression of SLC6A4 mRNA in adipose tissue compared to people with a normal BMI [42]. The DNMT3B gene (encoding DNA methyltransferase 3 beta, Q9UBC3) is not directly associated with obesity, but its involvement in DNA methylation can influence gene expression. It has been shown that knocking out this gene in brown fat in female mice promotes a thermogenic program, leading to increased energy expenditure and reduced obesity [43]. The ATP8A1 gene (encoding ATPase phospholipid transporting 8A1, Q9Y2Q0) encodes an enzyme that belongs to the P-type ATPases family. This enzyme is involved in the transport of phospholipids across cellular membranes. In patients with obesity, there was an increased expression of the ATP8A1 gene in visceral adipose tissue [44]. The CDK6 gene (cyclin-dependent kinase 6, Q00534) regulates biological pathways associated with the cell cycle. CDK inhibitors, traditionally known for their effectiveness in cancer therapy, are now emerging as a promising treatment for diet-induced obesity [45].

### 3.2. Proteomic Patterns

The proteomic profiling was performed as described previously in Kiseleva et al. [25]. In total, 708 proteins were identified, of which 46 were found in all samples. Proteins that were detected in 10 samples or fewer were filtered out. Thus, the final proteome matrix contained 101 samples and 174 protein features. 

Proteome data were used to build models for BMI prediction using the cross-validation procedure. A total of 100 runs of 10-fold cross-validation procedures were performed. The average prediction accuracy across all models was 5.44 ± 0.31 kg/m^2^ (mean ± 95% confidence interval).

Since the sample under study includes many patients with increased BMI, proteins with non-zero coefficients in the BMI prediction model can potentially participate in young obesity onset. Each model included 24 proteins on average, with the first quartile equal to 16 proteins and the third quartile equal to 36 proteins. Information on how many times each of the 174 proteins has been included in the sparse models is available in Supplementary Table S3.

#### 3.2.1. Key Proteins in Predictive Models

In our analysis, a set of 13 proteins was consistently present in over 80% of predictive models. These proteins have been extensively studied both individually and in relation to obesity and associated metabolic disorders. Proteins of interest are representatives of the serpins, complement system, apolipoproteins, and antioxidants. We offer a concise literature review summarizing the accumulated knowledge on the association of these proteins with obesity (see Supplementary Document S2).

#### 3.2.2. Overrepresentation of Certain Proteins

**Figure 3:**
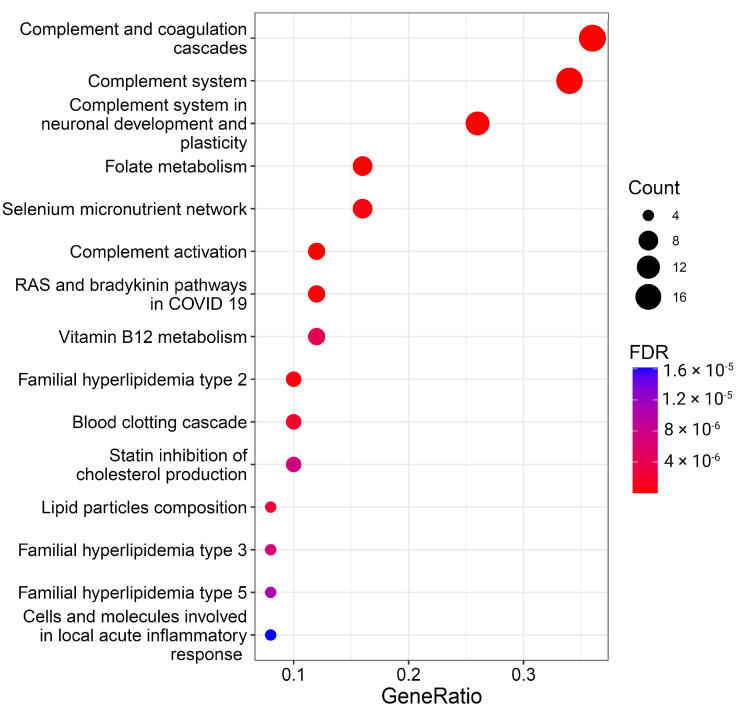
Overrepresentation analysis of protein BMI predictors. The geneRatio value (x-axis) is the proportion of genes in a specific function/pathway from the total differentially expressed genes. The dot size is proportional to the gene count enriched in the pathway. The color of the dot shows the significance of pathway enrichment. FDR—false discovery rate.

The meticulous examination of specific proteins lays the groundwork for exploring the overrepresentation of these proteins within molecular pathways, providing a comprehensive understanding of their functional roles and interactions in cellular processes. Thus, we performed enrichment analysis to find whether proteins selected as significant BMI predictors are overrepresented in known pathways. The results of the overrepresentation analysis are visualized in Figure 3 and Supplementary Table S4.

A total of 26 pathways with FDR < 0.05 were significantly enriched with BMI predictor proteins (Figure 3, Supplementary Table S4). While some pathways, especially those related to the complement system and lipids, are well-documented in the literature and this manuscript, the more captivating and less apparent results from the overrepresentation analysis spotlight folate, vitamin B12, and selenium metabolism, as well as RAS and bradykinin pathways.

Concerning folate, it is known that overweight and obese individuals exhibit lower serum folate concentrations compared to normal individuals, attributed to increased folic acid use, urinary excretion, blood volume dilution, tissue-specific variations, and changes in folate’s endocrine functions. Notably, lower folate intake and serum levels are independently associated with greater body weight and central adiposity [46,47]. Individuals with higher BMIs, characterized by lower supplement use and unhealthier diets, may experience reduced vegetable and fruit consumption, impacting folate levels. Additionally, adiposity may influence folate absorption by the intestinal epithelium [48].

Selenium, implicated in antioxidant processes, reproduction, and thyroid metabolism, experiences alterations in its nutritional status in conditions of excess adiposity, particularly a decrease in glutathione peroxidase activity in adults with obesity [49]. Selenium status in obesity, assessed through various biomarkers, reveals heterogeneous data from human and laboratory studies, with conflicting results on Se and biomarker levels. Discrepancies may arise from environmental exposure levels and Se species in studied populations [50]. There are some indications that young children with excess weight show an impaired selenium status [51].

The kallikrein–kinin system’s component, bradykinin, linked to glucose homeostasis, has demonstrated improvements in insulin action and glucose uptake in isolated muscle or fat cells upon acute stimulation. Being associated with inflammatory response and various vascular permeability functions, bradykinin-mediated signaling may be linked to obesity, diabetes, and other diseases [52,53]. 

Serum vitamin B12 concentrations’ association with obesity has yielded inconsistent findings; however, low vitamin B12 levels are associated with obesity and overweight [54], but not insulin resistance, metabolic syndrome, or gender [55].

### 3.3. Metabolome Analysis

We then turned to the analysis of metabolome data. In total, 313 metabolites were identified, of which 19 were found in all samples. Metabolites detected in 10 samples or fewer were filtered out, and the final metabolome matrix contained 101 samples and 108 features. 

Similar to proteome analysis, we utilized metabolome data to train 100 sparse PLS models for BMI prediction using the 10-fold cross-validation procedure. The average prediction accuracy across all models was 6.06 ± 0.33 kg/m^2^ (mean ± 95% confidence interval).

Each model included 38 metabolites on average, with the first quartile equal to two metabolites and the third quartile equal to 54 metabolites. Information on how many times each of the 313 metabolites has been included in the sparse models is available in Supplementary Table S5. We found that glutamic acid and cystine were included in at least 95% of all models, while other metabolites were included in fewer than 65%.

High levels of glutamic acid and cystine are associated with obesity [56]. These amino acids correlate with anthropometric parameters and markers of metabolic dysfunction, particularly triglyceride levels, high-density lipoprotein cholesterol, and the homeostasis model assessment of insulin resistance (HOMA-IR) [57,58,59]. Mouse experiments with cystine supplementation showed that high amino acid consumption reduces energy expenditure and increases fat mass. An analysis of mice on a high-cystine diet revealed that elevated amino acid consumption decreases energy expenditure, thereby increasing fat mass [60].

**Figure 4:**
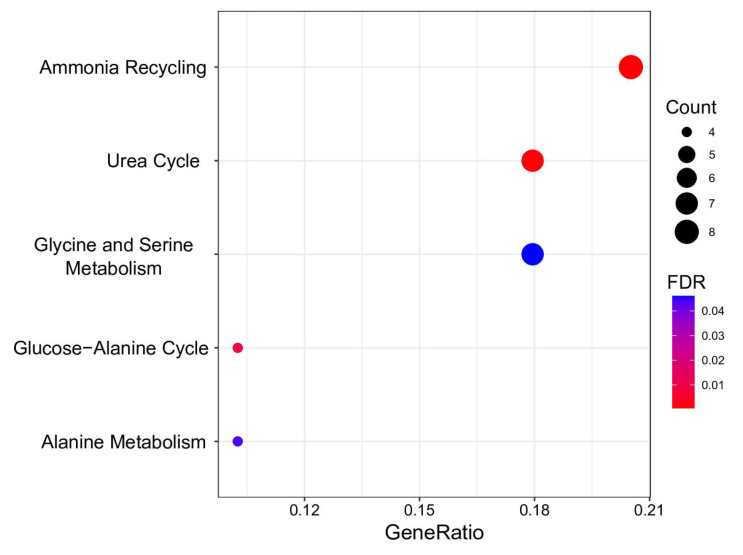
Overrepresentation analysis of metabolic BMI predictors. The geneRatio value (x-axis) is the proportion of genes in a specific function/pathway from the total differentially expressed genes. The dot size is based on the gene count enriched in the pathway, and the dot color shows the significance of pathway enrichment. FDR—false discovery rate.

For a more in-depth study, we analyzed whether significant metabolic BMI predictors were overrepresented in HMDB primary metabolic pathways. The results are visualized in Figure 4 and Supplementary Table S6.

Five pathways demonstrating FDR < 0.05 were discerned as significantly enriched with metabolites predictive of BMI. These pathways are pivotal in the biological system’s energy production, protein synthesis, and nitrogen exchange. Notably, “Ammonia Recycling” and the “Urea Cycle” exhibit intricate interconnections. The former involves reprocessing ammonia and its accrual in the organism, while the latter represents a critical stage in converting cytotoxic ammonia into urea, facilitating its safe elimination from the organism [61]. Perturbations in these pathways were noted in metabolically unhealthy young individuals with obesity [62]. 

Metabolites predictive of BMI were found to be associated with processes involving amino acid metabolism, encompassing “Glycine and Serine Metabolism”, “Glucose-alanine cycle”, and “Alanine metabolism”. Individuals with obesity displayed diminished levels of glycine and serine, potentially arising from a confluence of reduced intestinal absorption, diminished biosynthesis, and heightened catabolism or excretion in urine [63,64]. Diminished serine and glycine concentrations have been identified as potential indicators for discerning metabolically healthy adults from unhealthy adults (with more than two metabolic syndrome risk factors), even after adjusting for BMI [65]. The conversion of alanine to pyruvate in the liver establishes a linkage between the glucose–alanine cycle and alanine metabolism, which is imperative for maintaining stable blood glucose levels. Disruptions in these pathways have been documented in metabolic dysfunction associated with obesity [66]. Significantly, hyperglycemia, a characteristic feature of obesity, has been correlated with muscle atrophy [67,68].

### 3.4. Multiomics Analysis for BMI Prediction

**Figure 5:**
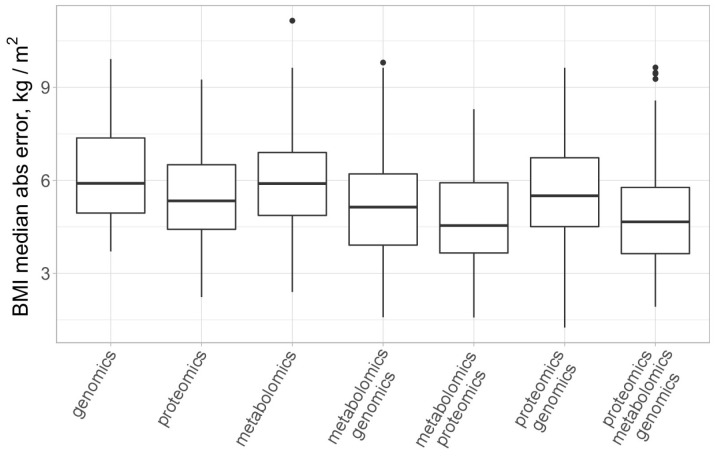
Comparison of several single and multiomics models for BMI prediction. All models employ sparse PLS regression and were trained via 100 runs of 10-fold cross-validation. Outliers are marked with black dots.

Finally, we turned to the multi-omics analysis of the data. For this purpose, we utilized the block.spls() function from the mixOmics package [34], which performs the so-called N-integration of multiple datasets measured on the same set of samples or observations with variable selection in each data set. Several sparse PLS models were trained to predict BMI for different combinations of plasma molecular analytes (proteomics and metabolomics) and genotyping results. A comparison of the model’s performance is presented in Figure 5.

We report that models trained on the combination of proteomics and metabolomics, on average, had higher accuracy for BMI prediction (MAE = 4.77 ± 0.33 kg/m^2^) as compared to models trained on metabolomics plus genomics data (MAE = 5.83 ± 0.37 kg/m^2^) and models trained on proteomics plus genomics data (MAE = 5.54 ± 0.31 kg/m^2^). For the “full” models, combining proteomics, metabolomics, and genomics data, we found that the average median absolute error for BMI prediction was equal to 5.08 ± 0.32 kg/m^2^. This is not significantly different from the performance of the dual-omics models trained on a combination of proteomics and metabolomics data according to the Wilcoxon test (*p* = 0.22).

The structure of associations between features from different omics layers was visualized in the form of the relevance network graph using the network() function from the mixOmics package. This technique may be seen as a robust approximation of the Pearson correlation since it borrows information from the projection of initial features to the latent variables.

**Figure 6:**
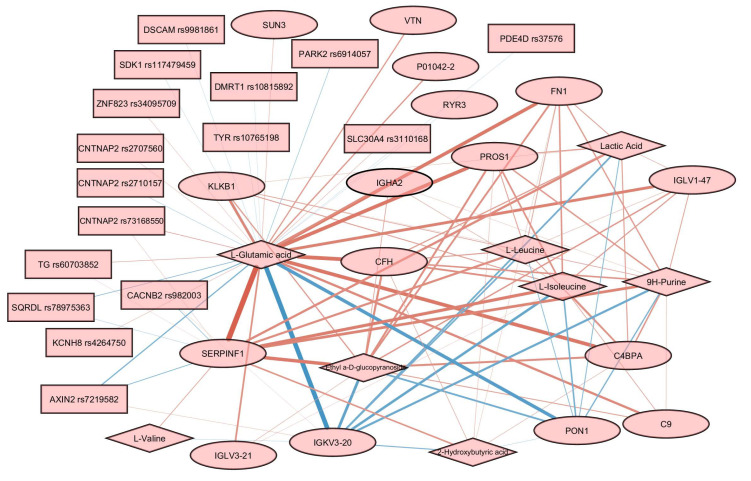
Relevance associations network connecting features from three omics layers. Proteins are represented by ellipses, metabolites by diamonds, and SNPs by rectangles. Edge width is proportional to the variable similarity. Red edges indicate a positive correlation. Blue edges indicate a negative correlation.

In order to build the relevance association network, we trained a sparse PLS model using all the available data for three omics layers. The final loadings for every omics layer are available in Supplementary Figure S1. The tuning threshold for the relevant associations’ network was set to 0.3 to reduce the visual clutter in the resulting graph. The obtained network contains 16 proteins, 8 metabolites, and 16 SNPs (Figure 6).

Glutamic acid was a hub in the graph, with edges connecting it to almost every node. The most prominent positive associations were found between glutamic acid and SERPINF1, and between glutamic acid and several other proteins, including PROS1, FN1, CFH, and C4BPA. Negative associations are reported for glutamic acid and several proteins, including IGKV3-20 and PON1. Associations involving SNPs are less prominent than associations involving proteins and metabolites. Several genes carrying SNPs were found to be associated with glutamic acid, including CNTNAP2, PDE4D, TG, and SDK1.

We did not find direct confirmation of the identified relationships in Figure 6 based on the available data on the processes, including these molecules. However, analysis of interactomes for highlighted proteins according to StringDB data showed that, for a quarter of them, there are enrichments for participation in glutamic acid regulation processes. The non-randomness of our results can also be confirmed by the discovered correlations (Figure 6) for 8 (SERPINF1, FN1, CFH, PDE4D, PROS1, CFHR5, CFH, and TG) of 16 highlighted proteins with glutamic acid and/or lactic acid, identified as part of the analysis of dependencies between proteins and metabolites, manifested in human blood plasma [69]. The authors of work [69] also compared their results with several characteristics, including BMI. As expected, the revision of the data to adjust for BMI did not change the associations. Thus, five out of seven proteins retained a correlation with glutamic acid and five out of eight with lactic acid (Supplementary Figure S2). According to the authors, the identified protein–metabolite relationships represent basic processes that are resistant to population fluctuations.

## 4. Discussion

Here, we report the results of a pilot study of molecular features associated with obesity in young adults. Multiomics profiling of blood plasma included SNP genotyping, semiquantitative proteomics, and metabolomics. As far as the authors know, this is the first multiomics study of obesity in the cohort of young adults with an equal proportion of men and women. Previous multiomic obesity research investigated individuals of diverse age ranges [14] or a study cohort exclusively comprised of female participants [24].

Our study systematically evaluated several single-omic and multiomic models for predicting BMI. Notably, our findings underscore the superior performance of proteome profiling of blood plasma among single-omic approaches, followed by metabolome profiling, while genomics exhibited the least favorable results. This outcome aligns with expectations, considering that the current polygenic risk scores, encompassing over two million variants, account for a mere 8.4% of the BMI variation, thereby contributing to the overall weak predictive capacity, as documented in previous studies leading to the weak predictive performance [20]. Simultaneously, the prominence of proteomics over metabolomics in BMI prediction aligns with findings from Watanabe et al. [14], although our study reveals a more pronounced superiority in this regard. These results shed light on the intricate interplay of molecular factors influencing BMI and emphasize the potential of proteomic signatures as biomarkers of obesity development. 

This study serves as a preliminary investigation, aiming to provide initial insights into the complex landscape of obesity. For example, a notable limitation lies in the homogeneity of our participant sample, which comprises exclusively Caucasian individuals. While this minimizes potential confounding variables related to genetic diversity, our findings may not be directly generalizable to other ethnic groups. Also, the omics techniques employed in our study—specifically, proteomics and metabolomics—come with analytical limitations. GC–MS (as well as two-dimensional gas chromatography coupled with mass spectrometry) has a reputation as one of the most reliable and robust analytical platforms for metabolomic studies, accompanied by a variety of spectral libraries for processing experimental data. This technique is becoming the new frontier for GC–MS-based metabolomics. It is used to characterize several classes of chemical compounds for panoramic and targeted studies of various biological samples, including blood plasma. However, despite being a powerful tool, without derivatization, gas chromatography only captures a subset of volatile and thermally stable metabolites [70]. Hence, molecular features retained in our omics-based BMI prediction models do not necessarily imply causality in the context of obesity. Establishing causal relationships requires rigorous validation and further verification on independent datasets. 

Our study lacks detailed information regarding participants’ dietary preferences. This gap prevents us from concluding the potential associations between obesity and specific diets (e.g., high protein, keto, or high glycemic index). Another beneficial addition would include microbiomics in the dataset and consideration for clinical assays. Furthermore, the absence of information regarding participants’ history of obesity treatment limits our ability to account for potential confounding variables. 

In an ideal scenario, a longitudinal study would capture the dynamic nature of obesity-related biomarkers in young adults over time. The temporal aspect is important for understanding the evolution of these biomarkers and their potential as indicators of obesity progression.

We employed sPLS models, recognized for their efficacy in integrating omics data by effectively handling high dimensionality, thereby facilitating the identification of key obesity-associated biomarkers. However, the accuracy of the obtained models is limited, with the best model achieving a median absolute error of 5.08 ± 0.32 kg/m^2^. We utilized the Multiomics Factor Analysis as an alternative to the supervised sPLS approach, which also did not show strong performance. However, numerous other advanced methods for integrating omics data remain unexplored. The modest performance of multiomics models may be explained by the sparsity of the available omics data: the final proteome and metabolome matrices contained only 174 and 108 features, respectively. Another possible explanation of suboptimal performance is the specific nature of our sample cohort, consisting of young adults. Supporting evidence for this assumption comes from our cohort, where age and gender did not emerge as predictors of BMI. Conversely, in other studies encompassing cohorts of all ages, it has been consistently demonstrated that BMI correlates with both gender and age [28] [PMC6441449]. It may turn out that BMI prediction for young adults is a more challenging task compared to the analysis of cohorts covering both young and old patients. 

In the present study, we intentionally did not focus on a comparative analysis between normal and obese patient groups. Our primary objective was not classification but rather regression analysis to understand the relationships between various omics features and the continuous measure of the phenotype of interest, BMI. Using regression instead of more traditional between-group comparison groups allowed us to maximize the sample size utilized, a crucial advantage when the sample size is limited. However, we note that group analysis may provide additional insights into obesity development in young adults. Addressing this and other limitations mentioned above will become a central focus for future research endeavors.

This study marks a crucial step forward in obesity research, offering a holistic view of its molecular landscape in the context of young individuals. By employing a multiomics approach, the research opens new avenues for future investigations. It underscores the importance of considering multiple layers of biological information to address the challenges posed by obesity comprehensively.

## 5. Conclusions

We identified a set of proteomic, metabolomic, and genomic biomarkers associated with obesity in young adults. Proteomics emerged as the most effective single-omics analysis, closely followed by metabolomics. The combination of these approaches in a multiomics model has demonstrated the highest level of performance in terms of BMI prediction. We hope that the obtained results and data will prove valuable to researchers in systems medicine to combat this socially significant disease.

## Figures and Tables

**Table 1:** Characteristics of patients enrolled in the study (mean ± standard deviation). Patient BMI cutoffs are given according to World Health Organization standards (underweight: <18.5 kg/m^2^, normal: 18.5–25 kg/m^2^, overweight: 25–30 kg/m^2^, obese: ≥30 kg/m^2^) [39].

	All	Underweight	Normal	Overweight	Obese
Number of patients	101	2	20	21	58
Male to female ratio	50/51	0/2	9/11	11/10	30/28
Age (years)	31.6 ± 6.7	29.0 ± 1.4	30.7 ± 5.6	32.9 ± 6.7	31.6 ± 7.2
BMI (kg/m^2^)	32.8 ± 9.2	17.8 ± 0.6	22.1 ± 1.5	27.5 ± 1.3	38.9 ± 7.0

## Data Availability

All datasets are available from the corresponding author upon request. Raw proteome data are publicly released on ProteomeXchange with the dataset identifier PXD023526. Raw metabolome data are publicly released on MetaboLights with the dataset identifier MTBLS8961. Genotyping data are publicly released on the Mendeley Data Portal with the dataset identifier 10.17632/mgg6cf4j64.1. Quality control of proteomic and metabolomics data is also available on the Mendeley Data Portal with the dataset identifier 10.17632/t255cjz787.1.

## Supplementary Materials

The following supporting information can be downloaded at: https://www.mdpi.com/article/10.3390/biology13040272/s1, Table S1: Anthropometrical and clinical characteristic of individuals under study; Table S2: Genes included in the sparse models; Table S3: Proteins include in the sparse models; Table S4: Results of the overrepresentation analysis for proteins; Table S5: Metabolites include in the sparse models; Table S6: Results of the overrepresentation analysis for metabolites; Document S1: Quality control for proteomic and metabolomics datasets; Document S2: Key omics features in predictive models; Figure S1: Loadings for metabolomics, proteomics, and genomics; Figure S2: Protein–metabolite correlations value from public data. References [71,72,73,74,75,76,77,78,79,80,81,82,83,84,85,86,87,88,89,90,91,92,93,94,95,96,97,98,99,100,101,102,103,104] are cited in the supplementary materials.
