# Supplementary material for: Multiomics Picture of Obesity in Young Adults
Source: Biology (Basel). 2024 Apr 18;13(4):272. doi: 10.3390/biology13040272 (PMC11048234; doi:10.3390/biology13040272)
Supplement: Supplementary file 1 [file biology-13-00272-s001.zip › Document_S1.pdf]

Supplement document S1: Quality control for proteomic and metabolomics dataset

Proteomics

As part of the proteomics experiment, to ensure proper data quality, we:

- calibrated the device (washed the external optics and injected calibration mixture),
- tested digested standards (HeLa cell line digest, cytochrome C) and blank samples,
- shuffled the acquisition sequence to minimize the batch effect,
- carried out a bioinformatic interpretation of all experimental results in a unified mode (one bioinformatic protocol with constant search parameters).

The results of data analysis performed in the PeptideShaker package provide an unbiased estimation of the quality of identified and semi-qualified hits, the validity of the mass spectrometer, and the search engines. These data are available in protein-, peptide- and PSM-centered modes for all samples and all technical replicates (available in Mendeley Data through dataset identifier 10.17632/t255cjz787.1).

Below, we provide examples of available plots for each technical run. These plots allow inspection and fine-tuning of the validation process and examination of the results' quality.

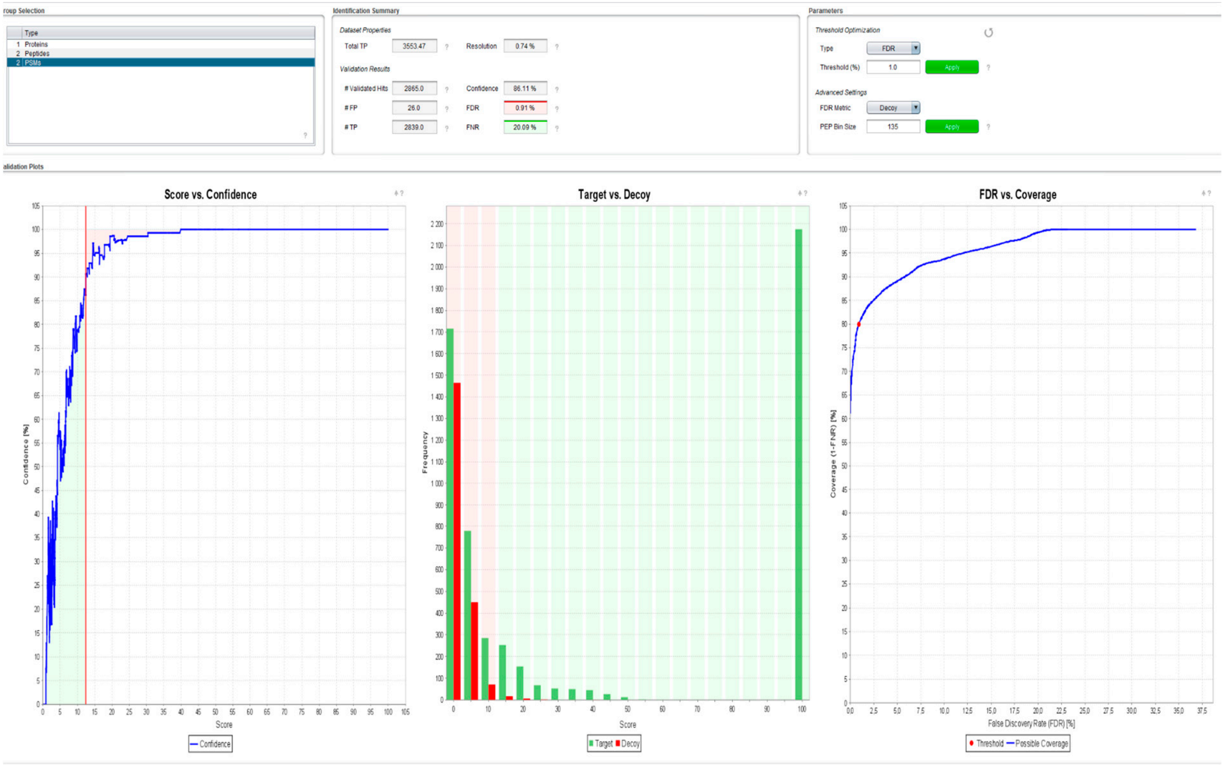

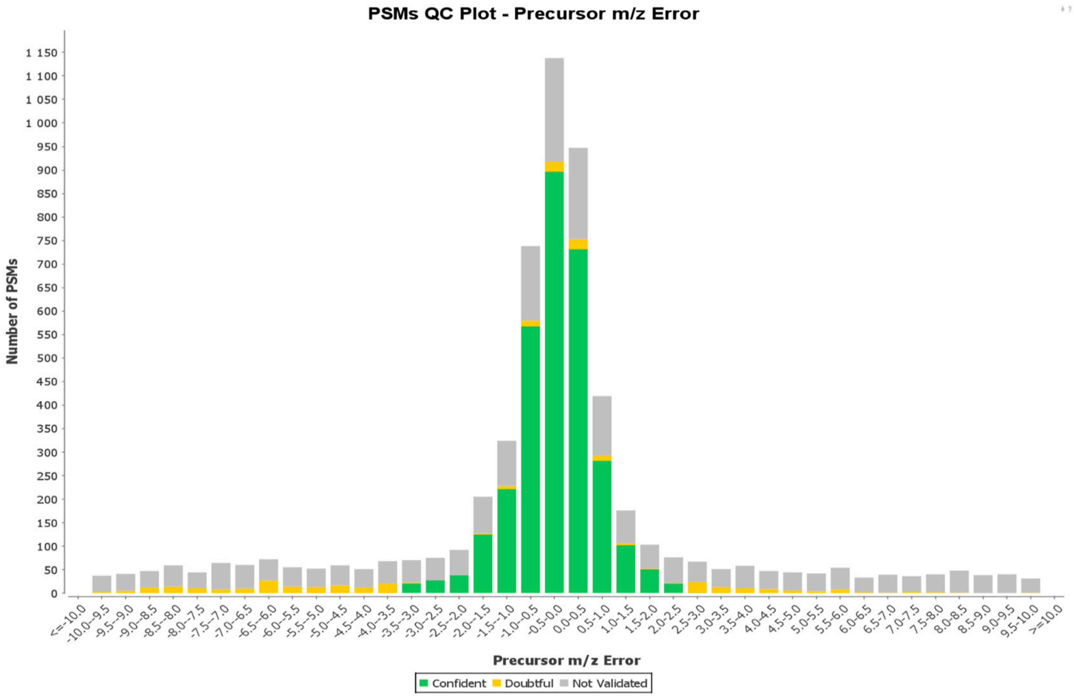

Metabolomics

For metabolomics, our quality control included several stages:

- autotune/PFTBA mass calibration,
- injections of blank samples (pyridine, method/reagent blank),
- using FAME standards to minimize biological matrix effects and monitor column degradation.

The most appropriate solution contains analytes distributed as fully as possible across the m/z and retention time ranges to assess the full analysis window. The results were evaluated for the mass-to-charge (m/z) ratio and chromatographic characteristics, including retention time, peak area, and peak shape (e.g., tailing factor), and compared to pre-defined acceptance criteria.

For our dataset (estimated for FAME standards), we maintained:

- m/z error of 0.01 Da compared to theoretical mass,
- retention time error of < 1% compared to the defined retention time,
- peak area equal to a predefined acceptable peak area ± 10%,
- symmetrical peak shape without peak splitting.

We obtained metabolomics data as part of this project and are publishing it here for the first time. We are pleased to demonstrate box plot, which provide the locality, spread, and skewness of chromatographic parameters for ten FAMEs.

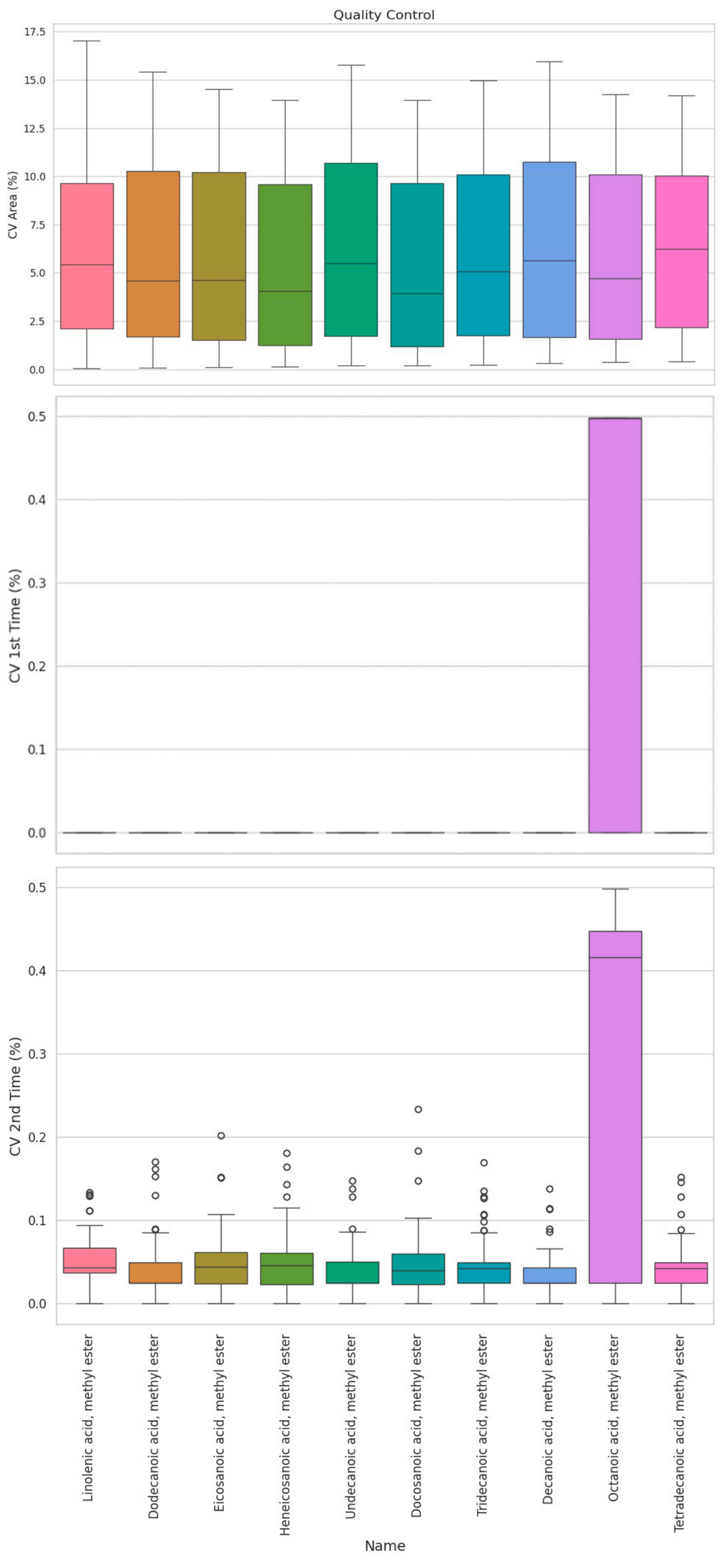

Quality Control
CV Area (%)
17.5
15.0
12.5
10.0
7.5
5.0
2.5
0.0
CV 1st Time (%)
0.5
0.4
0.3
0.2
0.1
0.0
CV 2nd Time (%)
0.5
0.4
0.3
0.2
0.1
0.0
Linolenic acid, methyl ester
Dodecanoic acid, methyl ester
Eicosanoic acid, methyl ester
Heneicosanoic acid, methyl ester
Undecanoic acid, methyl ester
Docosanoic acid, methyl ester
Tridecanoic acid, methyl ester
Decanoic acid, methyl ester
Octanoic acid, methyl ester
Tetradecanoic acid, methyl ester
Name
