# Supplementary material for: Multiomics Picture of Obesity in Young Adults
Source: Biology (Basel). 2024 Apr 18;13(4):272. doi: 10.3390/biology13040272 (PMC11048234; doi:10.3390/biology13040272)
Supplement: Supplementary file 1 [file biology-13-00272-s001.zip › Document_S2.pdf]

Supplementary document S2: Key omics features in predictive models

Unsupervised approach to omics analysis

In order to identify omics features serving as predictors for BMI and to conduct multi-omics analysis, we employed sparse Partial Least Squares regression — a supervised method that transforms thousands of omics features into a new low-dimensional space of latent components. As an alternative to the supervised approach, we adopted the Multi-Omics Factor Analysis (MOFA), which operates within a factor analysis model framework designed for integrating multi-omic datasets. Similar to Principal Component Analysis, MOFA infers an interpretable low-dimensional representation in terms of a few latent factors [1] and does not require training data.

We used the MOFA2 R package to analyze omics data from obese and healthy patients. Each individual omics dataset, as well as every combination of omics datasets, served as input for the MOFA method, utilizing default settings. The latent factors identified through the MOFA method were employed to categorize patients into four groups (underweight, normal, overweight, obese) using the Partitioning Around Medoids (PAM) method. A qualitative assessment was conducted to evaluate how the obtained sample clustering corresponded to the ground truth, employing the adjusted Rand index. Results are presented in the Table below.

Dataset	Adjusted Rand index
Proteomics	0.120
Metabolomics	0.038
Genomics	0.003
Proteomics + Metabolomics	-0.030
Metabolomics + Genomics	0.003
Proteomics + Genomics	-0.007
Proteomics + Metabolomics + Genomics	0.003

We report that according to unsupervised approach to factor analysis via MOFA, proteomics data were the best predictors of patient clustering (adjusted Rand index R=0.12) as compared to metabolomics (adjusted Rand index = 0.038). This confirms our findings for the supervised approach performed using the supervised method (sPLS). However for genomics, as well as for any multiomics combinations, latent factors identified via MOFA failed to cluster patients into obesity-related groups with adjusted Rand indexes almost equal to zero.

Key proteins in predictive models

Serpins: SERPINA1 and SERPINF1 encoded proteins

Serpins are a class of proteins that act as inhibitors of chymotrypsin-like serine proteases. Two notable members of this protein family, alpha-1-antitrypsin (P01009) and pigment epithelium-derived factor (P36955), have been implicated in the context of obesity. Reduced levels of alpha-1-antitrypsin in the blood serum, coupled with elevated levels of neutrophil elastase (which is inhibited by alpha-1-antitrypsin) in adipocytes, contribute to obesity development. Alpha-1-antitrypsin anomalies are associated with

chronic obesity-related inflammation, adipose tissue remodeling, insulin resistance, and liver steatosis [2].

Similarly, pigment epithelium-derived factor, glycoproteins abundantly secreted by adipocytes, is suggested as a potential mediator of obesity-induced insulin resistance [3]. Proteomic studies have indicated a P36955 as a potential marker of inflammation [3,4].

Complement system: C7, C9, CFH, and C4BPA encoded proteins

Intensified inflammation is implicated in cardiometabolic conditions. Initially perceived as an antimicrobial defense, the complement system's evolving role includes immune complex clearance, tissue regeneration, and metabolic regulation [5].

Complement gene perturbations and tissue remodeling are observed in obesity and non-alcoholic fatty liver disease (NAFLD). Specifically, C9 gene expression (P02748) decreases across NAFLD stages, with reduced C9 in obesity and nonalcoholic steatohepatitis [6,7] Chronic elevation of C7 (P10643) is also associated with metabolic syndrome and obesity [8].

Complement factor H (P08603, fH) maintains complement homeostasis and restricts complement action to activating surfaces. Accelerated alternative pathway activation, facilitated by fH, is suggested as a link between obesity and metabolic disorders [9–11].

C4-binding protein, a large and abundant plasma glycoprotein, increases its alpha-chain isoform during inflammation. Alpha-chain (P04003) controls the classical pathway of complement activation and possesses binding sites for various molecules (serum amyloids, heparin, low-density lipoprotein receptor-related protein, and bacterial surfaces). This multifaceted binding capability underscores the significance of C4BPA in inflammation, lipid metabolism, and coagulation pathways. Deviations in C4BPA expression are linked to obesity [12,13], including obesity [14], highlighting its potential as a therapeutic target for obesity-related pathologies.

Apolipoproteins: APOD and APOA4 encoded proteins

Apolipoproteins are pivotal in obesity, impacting lipid metabolism, energy expenditure, and inflammation—key factors in obesity development [15].

ApoA4 (P06727), the third most abundant apolipoprotein in high-density lipoproteins, promotes cholesterol efflux, thermogenesis, and insulin sensitivity, thereby reduces inflammation. With anti-oxidative and anti-inflammatory properties, ApoA4 potentially contributes to cardiovascular protection. Fasting plasma ApoA4 levels are higher in obese individuals than their lean counterparts, [16]. On the other hand, Post-Roux-en-Y gastric bypass surgery leads to a significant increase in ApoA4 levels due to its impact on gastric emptying and satiety [17].

The situation is even more intricate with ApoD (P05090), which does not share significant homology with canonical apolipoproteins and is structurally similar to lipocalins responsible for transporting lipids. Its anti-inflammatory function is still under exploration. However, the upregulation of the ApoD gene has been linked to decreased oxidative stress and inflammation in various pathologies, including Alzheimer's disease, Parkinson's disease, and cancer. High levels of ApoD have also been associated with better metabolic health in morbidly obese women. Impressive research [18] using a

mouse model has allowed for speculation on potential mechanisms by which ApoD regulates body weight and energy homeostasis through increased energy expenditure, although further evidence is needed from other biological models. Additionally, overexpression of ApoD in the hypothalamus has been found to cause leptin resistance without manifesting weight gain or hyperphagia [19].

Furthermore, the study in fruit flies suggested ApoD’s link to anti-oxidation and anti-stress activities, contributing to lifespan expansion [20]. In young-onset obesity, ApoD's association with age and neurological health suggests potential roles in lipid metabolism and aging [20].

Antioxidants: GPX3 and PON1 encoded protein

Oxidative stress, implicated in obesity, forms a feedback loop affecting white adipose tissue deposition and food intake [21]. Genomic analysis revealed associations between mutations in antioxidant enzyme families GPx and PON and obesity risk in a pediatric cohort. This observation is supported by disrupted serum concentrations of PON1 (P27169) and GPX3 (P22352) are disrupted in cases of human obesity and metabolic disorders [22,23].

PROS1 encoded protein

Protein S (P07225), a non-enzymatic cofactor for activated protein C, aids in inactivating pro-coagulant factors. Present in blood plasma in free and C4-binding protein complexes [24], it sparks interest in understanding obesity-associated metabolic aberrations. A diet efficacy study suggested it as a potential biomarker for successful weight maintenance under a high protein/low glycemic index diet [25].

In a study involving Japanese middle-aged obese women, total and free protein S antigen levels correlated with key biological molecules (e.g., triglycerides, total cholesterol, protein C), while no correlation was observed with BMI, visceral fat, and blood pressure [26].

A2M encoded protein

Alpha-2-macroglobulin (P01023) is a unique protein interacting with various endopeptidases. As a substrate for endopeptidases, P01023 lures active proteases into molecular cage for elimination. Besides regulating proteolysis, it contributes to immunity [27]. With highly conserved and widespread evolutionary properties, P01023 is implicated in various diseases. Elevated plasma levels of P01023 are associated with obesity, type 2 diabetes, chronic viral hepatitis, and various metabolic conditions, including dyslipidemia and steatosis [28,28,29].

AHSG encoded protein

Fetuin A (P02765) is a versatile plasma protein that acts as an insulin receptor tyrosine kinase inhibitor and indicates liver function. Its association with metabolic syndrome and type 2 diabetes complications is well-documented [30,31]. While fetuin A's correlation with BMI remains unclear [32,33], there are studies highlighting links between elevated levels of P02765, BMI and waist circumference [34].

References

1. Argelaguet, R.; Velten, B.; Arnol, D.; Dietrich, S.; Zenz, T.; Marioni, J.C.; Buettner, F.; Huber, W.; Stegle, O. Multi-Omics Factor Analysis-a Framework for Unsupervised Integration of Multi-Omics Data Sets. *Mol Syst Biol* **2018**, *14*, e8124, doi:10.15252/msb.20178124.
2. Mansuy-Aubert, V.; Zhou, Q.L.; Xie, X.; Gong, Z.; Huang, J.-Y.; Khan, A.R.; Aubert, G.; Candelaria, K.; Thomas, S.; Shin, D.-J.; et al. Imbalance between Neutrophil Elastase and Its Inhibitor A1-Antitrypsin in Obesity Alters Insulin Sensitivity, Inflammation, and Energy Expenditure. *Cell Metabolism* **2013**, *17*, 534–548, doi:10.1016/j.cmet.2013.03.005.
3. Böhm, A.; Ordelheide, A.-M.; Machann, J.; Heni, M.; Ketterer, C.; Machicao, F.; Schick, F.; Stefan, N.; Fritsche, A.; Häring, H.-U.; et al. Common Genetic Variation in the SERPINF1 Locus Determines Overall Adiposity, Obesity-Related Insulin Resistance, and Circulating Leptin Levels. *PLoS One* **2012**, *7*, e34035, doi:10.1371/journal.pone.0034035.
4. Geyer, P.E.; Wewer Albrechtsen, N.J.; Tyanova, S.; Grassl, N.; Iepsen, E.W.; Lundgren, J.; Madsbad, S.; Holst, J.J.; Torekov, S.S.; Mann, M. Proteomics Reveals the Effects of Sustained Weight Loss on the Human Plasma Proteome. *Mol Syst Biol* **2016**, *12*, 901, doi:10.15252/msb.20167357.
5. Shim, K.; Begum, R.; Yang, C.; Wang, H. Complement Activation in Obesity, Insulin Resistance, and Type 2 Diabetes Mellitus. *World J Diabetes* **2020**, *11*, 1–12, doi:10.4239/wjd.v11.i1.1.
6. Rensen, S.S.; Slaats, Y.; Driessen, A.; Peutz-Kootstra, C.J.; Nijhuis, J.; Steffensen, R.; Greve, J.W.; Buurman, W.A. Activation of the Complement System in Human Nonalcoholic Fatty Liver Disease. *Hepatology* **2009**, *50*, 1809–1817, doi:10.1002/hep.23228.
7. Subudhi, S.; Drescher, H.K.; Dichtel, L.E.; Bartsch, L.M.; Chung, R.T.; Hutter, M.M.; Gee, D.W.; Meireles, O.R.; Witkowski, E.R.; Gelrud, L.; et al. Distinct Hepatic Gene-Expression Patterns of NAFLD in Patients With Obesity. *Hepatol Commun* **2021**, *6*, 77–89, doi:10.1002/hep4.1789.
8. Gabrielsson, B.G.; Johansson, J.M.; Lönn, M.; Jernås, M.; Olbers, T.; Peltonen, M.; Larsson, I.; Lönn, L.; Sjöström, L.; Carlsson, B.; et al. High Expression of Complement Components in Omental Adipose Tissue in Obese Men. *Obes Res* **2003**, *11*, 699–708, doi:10.1038/oby.2003.100.
9. King, B.C.; Blom, A.M. Complement in Metabolic Disease: Metaflammation and a Two-Edged Sword. *Semin Immunopathol* **2021**, *43*, 829–841, doi:10.1007/s00281-021-00873-w.
10. Moreno-Navarrete, J.M.; Martínez-Barricarte, R.; Catalán, V.; Sabater, M.; Gómez-Ambrosi, J.; Ortega, F.J.; Ricart, W.; Blüher, M.; Frühbeck, G.; Rodríguez de Cordoba, S.; et al. Complement Factor H Is Expressed in Adipose Tissue in Association With Insulin Resistance. *Diabetes* **2010**, *59*, 200–209, doi:10.2337/db09-0700.
11. Li, J.; Shen, Y.; Tian, H.; Xie, S.; Ji, Y.; Li, Z.; Lu, J.; Lu, H.; Liu, B.; Liu, F. The Role of Complement Factor H in Gestational Diabetes Mellitus and Pregnancy. *BMC Pregnancy and Childbirth* **2021**, *21*, 562, doi:10.1186/s12884-021-04031-w.

12. Liu, X.; Jiang, C.; Yang, P. Association of Single Nucleotide Polymorphisms in the 5′ Upstream Region of the C4BPA Gene with Essential Hypertension in a Northeastern Han Chinese Population. *Mol Med Rep* **2017**, *16*, 1289–1297, doi:10.3892/mmr.2017.6736.
13. Ermert, D.; Blom, A.M. C4b-Binding Protein: The Good, the Bad and the Deadly. Novel Functions of an Old Friend. *Immunology Letters* **2016**, *169*, 82–92, doi:10.1016/j.imlet.2015.11.014.
14. Chang, C.-Y.; Tung, Y.-T.; Lin, Y.-K.; Liao, C.-C.; Chiu, C.-F.; Tung, T.-H.; Shabrina, A.; Huang, S.-Y. Effects of Caloric Restriction with Protein Supplementation on Plasma Protein Profiles in Middle-Aged Women with Metabolic Syndrome—A Preliminary Open Study. *J Clin Med* **2019**, *8*, 195, doi:10.3390/jcm8020195.
15. Natali, L.; Cavallini, A.; Cremonini, R.; Bassi, P.; Cionini, P.G. Amplification of Nuclear DNA Sequences during Induced Plant Cell Dedifferentiation. *Cell Differentiation* **1986**, *18*, 157–161, doi:10.1016/0045-6039(86)90081-3.
16. Ferrer, F.; Nazih, H.; Zaïr, Y.; Krempf, M.; Bard, J.M. Postprandial Changes in the Distribution of Apolipoprotein AIV between Apolipoprotein B- and Non Apolipoprotein B-Containing Lipoproteins in Obese Women. *Metabolism* **2003**, *52*, 1537–1541, doi:10.1016/j.metabol.2003.07.004.
17. Culnan, D.M.; Cooney, R.N.; Stanley, B.; Lynch, C.J. Apolipoprotein A-IV, a Putative Satiety/Antiatherogenic Factor, Rises after Gastric Bypass. *Obesity (Silver Spring)* **2009**, *17*, 46–52, doi:10.1038/oby.2008.428.
18. Jiménez-Palomares, M.; Cózar-Castellano, I.; Ganfornina, M.D.; Sánchez, D.; Perdomo, G. Genetic Deficiency of Apolipoprotein D in the Mouse Is Associated with Nonfasting Hypertriglyceridemia and Hyperinsulinemia. *Metabolism* **2011**, *60*, 1767–1774, doi:10.1016/j.metabol.2011.04.013.
19. Marcelin, G.; Gautier, E.L.; Clément, K. Adipose Tissue Fibrosis in Obesity: Etiology and Challenges. *Annu Rev Physiol* **2022**, *84*, 135–155, doi:10.1146/annurev-physiol-060721-092930.
20. Sanchez, D.; López-Arias, B.; Torroja, L.; Canal, I.; Wang, X.; Bastiani, M.J.; Ganfornina, M.D. Loss of Glial Lazarillo, a Homolog of Apolipoprotein D, Reduces Lifespan and Stress Resistance in Drosophila. *Curr Biol* **2006**, *16*, 680–686, doi:10.1016/j.cub.2006.03.024.
21. Manna, P.; Jain, S.K. Obesity, Oxidative Stress, Adipose Tissue Dysfunction, and the Associated Health Risks: Causes and Therapeutic Strategies. *Metab Syndr Relat Disord* **2015**, *13*, 423–444, doi:10.1089/met.2015.0095.
22. Adhe-Rojekar, A.; Mogarekar, M.R.; Rojekar, M.V. Paraoxonase Activity in Metabolic Syndrome in Children and Adolescents. *Caspian J Intern Med* **2018**, *9*, 116–120, doi:10.22088/cjim.9.2.116.
23. Çayır, A.; Turan, M.İ.; Gurbuz, F.; Kurt, N.; Yildirim, A. The Effect of Lifestyle Change and Metformin Therapy on Serum Arylesterase and Paraoxonase Activity in Obese Children. *J Pediatr Endocrinol Metab* **2015**, *28*, 551–556, doi:10.1515/jpem-2013-0486.
24. Dahlbäck, B. Protein S and C4b-Binding Protein: Components Involved in the Regulation of the Protein C Anticoagulant System. *Thromb Haemost* **1991**, *66*, 49–61.

25. Rubio-Aliaga, I.; Marvin-Guy, L.F.; Wang, P.; Wagniere, S.; Mansourian, R.; Fuerholz, A.; Saris, W.H.M.; Astrup, A.; Mariman, E.C.M.; Kussmann, M. Mechanisms of Weight Maintenance under High- and Low-Protein, Low-Glycaemic Index Diets. *Mol Nutr Food Res* **2011**, *55*, 1603–1612, doi:10.1002/mnfr.201100081.
26. Otsuka, Y.; Ueda, M.; Nakazono, E.; Tsuda, T.; Jin, X.; Noguchi, K.; Sata, S.; Miyazaki, H.; Abe, S.; Imai, K.; et al. Relationship between Plasma Protein S Levels and Apolipoprotein C-II in Japanese Middle-Aged Obese Women and Young Nonobese Women. *Blood Coagul Fibrinolysis* **2018**, *29*, 39–47, doi:10.1097/MBC.0000000000000662.
27. Vandooren, J.; Itoh, Y. Alpha-2-Macroglobulin in Inflammation, Immunity and Infections. *Front Immunol* **2021**, *12*, 803244, doi:10.3389/fimmu.2021.803244.
28. Netanel, C.; Goitein, D.; Rubin, M.; Kleinbaum, Y.; Katsherginsky, S.; Hermon, H.; Tsaraf, K.; Tachlytski, I.; Herman, A.; Safran, M.; et al. The Impact of Bariatric Surgery on Nonalcoholic Fatty Liver Disease as Measured Using Non-Invasive Tests. *The American Journal of Surgery* **2021**, *222*, 214–219, doi:10.1016/j.amjsurg.2020.11.045.
29. Deckmyn, O.; Poynard, T.; Bedossa, P.; Paradis, V.; Peta, V.; Pais, R.; Ratziu, V.; Thabut, D.; Brzustowski, A.; Gautier, J.-F.; et al. Clinical Interest of Serum Alpha-2 Macroglobulin, Apolipoprotein A1, and Haptoglobin in Patients with Non-Alcoholic Fatty Liver Disease, with and without Type 2 Diabetes, before or during COVID-19. *Biomedicines* **2022**, *10*, 699, doi:10.3390/biomedicines10030699.
30. Dabrowska, A.M.; Tarach, J.S.; Wojtysiak-Duma, B.; Duma, D. Fetuin-A (AHSG) and Its Usefulness in Clinical Practice. Review of the Literature. *Biomed Pap Med Fac Univ Palacky Olomouc Czech Repub* **2015**, *159*, 352–359, doi:10.5507/bp.2015.018.
31. Stefan, N.; Fritsche, A.; Weikert, C.; Boeing, H.; Joost, H.-G.; Häring, H.-U.; Schulze, M.B. Plasma Fetuin-A Levels and the Risk of Type 2 Diabetes. *Diabetes* **2008**, *57*, 2762–2767, doi:10.2337/db08-0538.
32. Stefan, N.; Hennige, A.M.; Staiger, H.; Machann, J.; Schick, F.; Kröber, S.M.; Machicao, F.; Fritsche, A.; Häring, H.-U. Alpha2-Heremans-Schmid Glycoprotein/Fetuin-A Is Associated with Insulin Resistance and Fat Accumulation in the Liver in Humans. *Diabetes Care* **2006**, *29*, 853–857, doi:10.2337/diacare.29.04.06.dc05-1938.
33. Kaushik, S.V.; Plaisance, E.P.; Kim, T.; Huang, E.Y.; Mahurin, A.J.; Grandjean, P.W.; Mathews, S.T. Extended-Release Niacin Decreases Serum Fetuin-A Concentrations in Individuals with Metabolic Syndrome. *Diabetes Metab Res Rev* **2009**, *25*, 427–434, doi:10.1002/dmrr.967.
34. Brix, J.M.; Stingl, H.; Höllerl, F.; Schernthaner, G.H.; Kopp, H.-P.; Schernthaner, G. Elevated Fetuin-A Concentrations in Morbid Obesity Decrease after Dramatic Weight Loss. *J Clin Endocrinol Metab* **2010**, *95*, 4877–4881, doi:10.1210/jc.2010-0148.
