# Supplementary material for: Multiomics Picture of Obesity in Young Adults
Source: Biology (Basel). 2024 Apr 18;13(4):272. doi: 10.3390/biology13040272 (PMC11048234; doi:10.3390/biology13040272)
Supplement: Supplementary file 1 [file biology-13-00272-s001.zip › Figure S1.pdf]

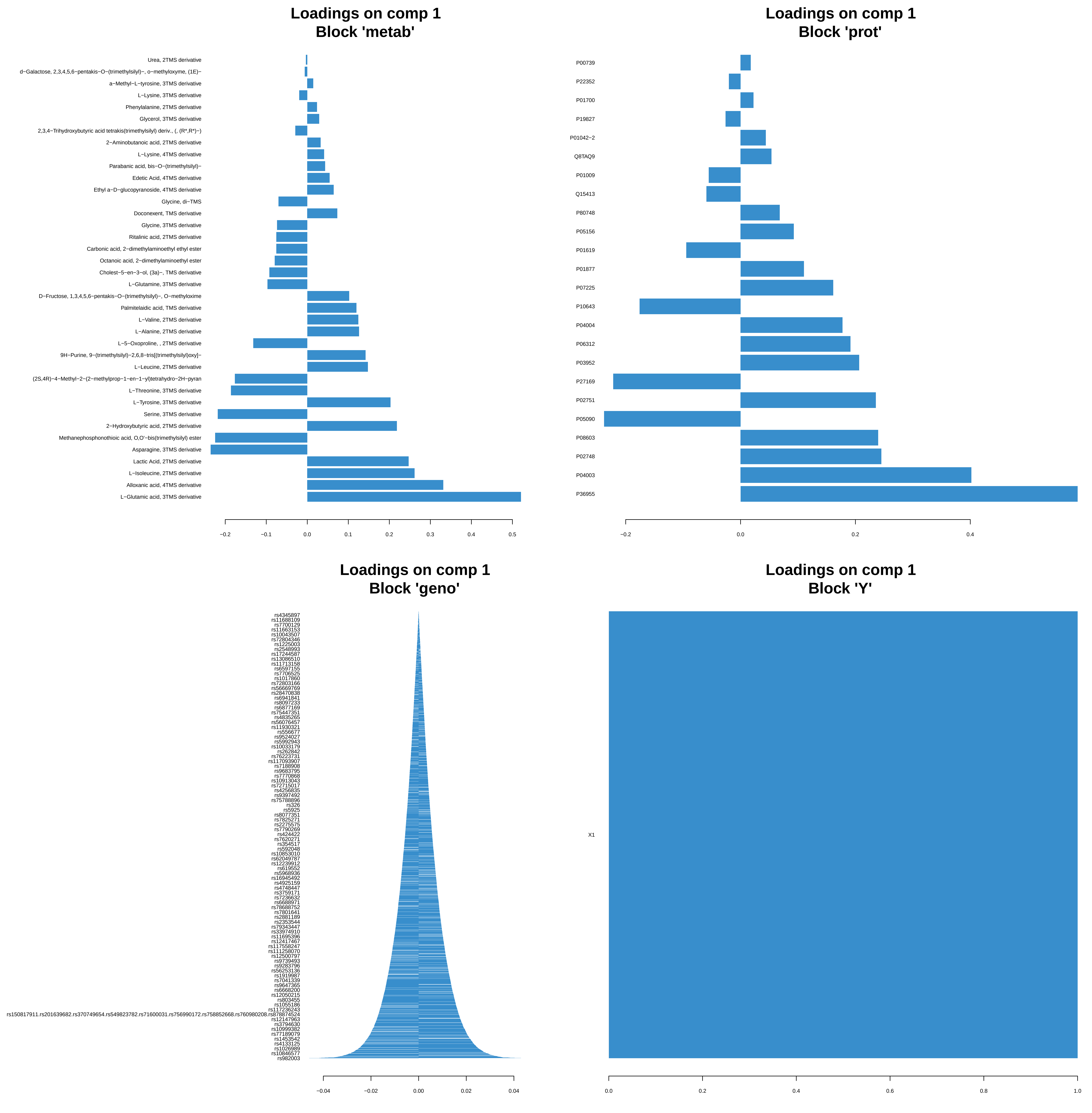
Loadings on comp 1
Block 'metab'
Urea, 2TMS derivative
d−Galactose, 2,3,4,5,6−pentakis−O−(trimethylsilyl)−, o−methyloxyme, (1E)−
a−Methyl−L−tyrosine, 3TMS derivative
L−Lysine, 3TMS derivative
Phenylalanine, 2TMS derivative
Glycerol, 3TMS derivative
2,3,4−Trihydroxybutyric acid tetrakis(trimethylsilyl) deriv., (, (R*,R*)−)
2−Aminobutanoic acid, 2TMS derivative
L−Lysine, 4TMS derivative
Parabanic acid, bis−O−(trimethylsilyl)−
Edetic Acid, 4TMS derivative
Ethyl a−D−glucopyranoside, 4TMS derivative
Glycine, di−TMS
Doconexent, TMS derivative
Glycine, 3TMS derivative
Ritalinic acid, 2TMS derivative
Carbonic acid, 2−dimethylaminoethyl ethyl ester
Octanoic acid, 2−dimethylaminoethyl ester
Cholest−5−en−3−ol, (3a)−, TMS derivative
L−Glutamine, 3TMS derivative
D−Fructose, 1,3,4,5,6−pentakis−O−(trimethylsilyl)−, O−methyloxime
Palmitelaidic acid, TMS derivative
L−Valine, 2TMS derivative
L−Alanine, 2TMS derivative
L−5−Oxoproline, , 2TMS derivative
9H−Purine, 9−(trimethylsilyl)−2,6,8−tris[(trimethylsilyl)oxy]−
L−Leucine, 2TMS derivative
(2S,4R)−4−Methyl−2−(2−methylprop−1−en−1−yl)tetrahydro−2H−pyran
L−Threonine, 3TMS derivative
L−Tyrosine, 3TMS derivative
Serine, 3TMS derivative
2−Hydroxybutyric acid, 2TMS derivative
Methanephosphonothioic acid, O,O'−bis(trimethylsilyl) ester
Asparagine, 3TMS derivative
Lactic Acid, 2TMS derivative
L−Isoleucine, 2TMS derivative
Alloxanic acid, 4TMS derivative
L−Glutamic acid, 3TMS derivative
−0.2 −0.1 0.0 0.1 0.2 0.3 0.4 0.5
Loadings on comp 1
Block 'prot'
P00739
P22352
P01700
P19827
P01042−2
Q8TAQ9
P01009
Q15413
P80748
P05156
P01619
P01877
P07225
P10643
P04004
P06312
P03952
P27169
P02751
P05090
P08603
P02748
P04003
P36955
−0.2 0.0 0.2 0.4
Loadings on comp 1
Block 'geno'
rs4345897
rs11688109
rs7700129
rs11663153
rs10043507
rs72804346
rs1225003
rs2548993
rs17244587
rs13086510
rs11713158
rs6597155
rs7706525
rs1017860
rs72803166
rs56669769
rs28470838
rs6941841
rs8097233
rs6877169
rs75447351
rs4835265
rs56076457
rs11930321
rs556677
rs9524027
rs5992943
rs10033179
rs262842
rs76223731
rs117093907
rs7188908
rs9683795
rs7770868
rs10913043
rs72715017
rs4256835
rs9397492
rs75788896
rs326
rs5925
rs8077351
rs7825271
rs2275575
rs7790269
rs424422
rs7620271
rs354517
rs592048
rs10853010
rs62049787
rs12239912
rs619552
rs5968936
rs16945492
rs4925159
rs4748447
rs3759171
rs7236632
rs6688971
rs78688752
rs7801641
rs2881189
rs2353544
rs79343447
rs33974910
rs11695396
rs12417467
rs117558247
rs111258070
rs12500797
rs9739493
rs9283796
rs56253136
rs1919987
rs7041339
rs9647365
rs6668200
rs12050215
rs803455
rs1055186
rs117236243
rs150817911.rs201639682.rs370749654.rs549823782.rs71600031.rs756990172.rs758852668.rs760980208.rs878874524
rs12147963
rs3794630
rs10999382
rs77189079
rs1453542
rs4133125
rs1026989
rs10846577
rs982003
−0.04 −0.02 0.00 0.02 0.04
Loadings on comp 1
Block 'Y'
X1
0.0 0.2 0.4 0.6 0.8 1.0
