# Supplementary figures and images for: Multiomics Picture of Obesity in Young Adults
Source: Biology (Basel). 2024 Apr 18;13(4):272. doi: 10.3390/biology13040272 (PMC11048234; doi:10.3390/biology13040272)

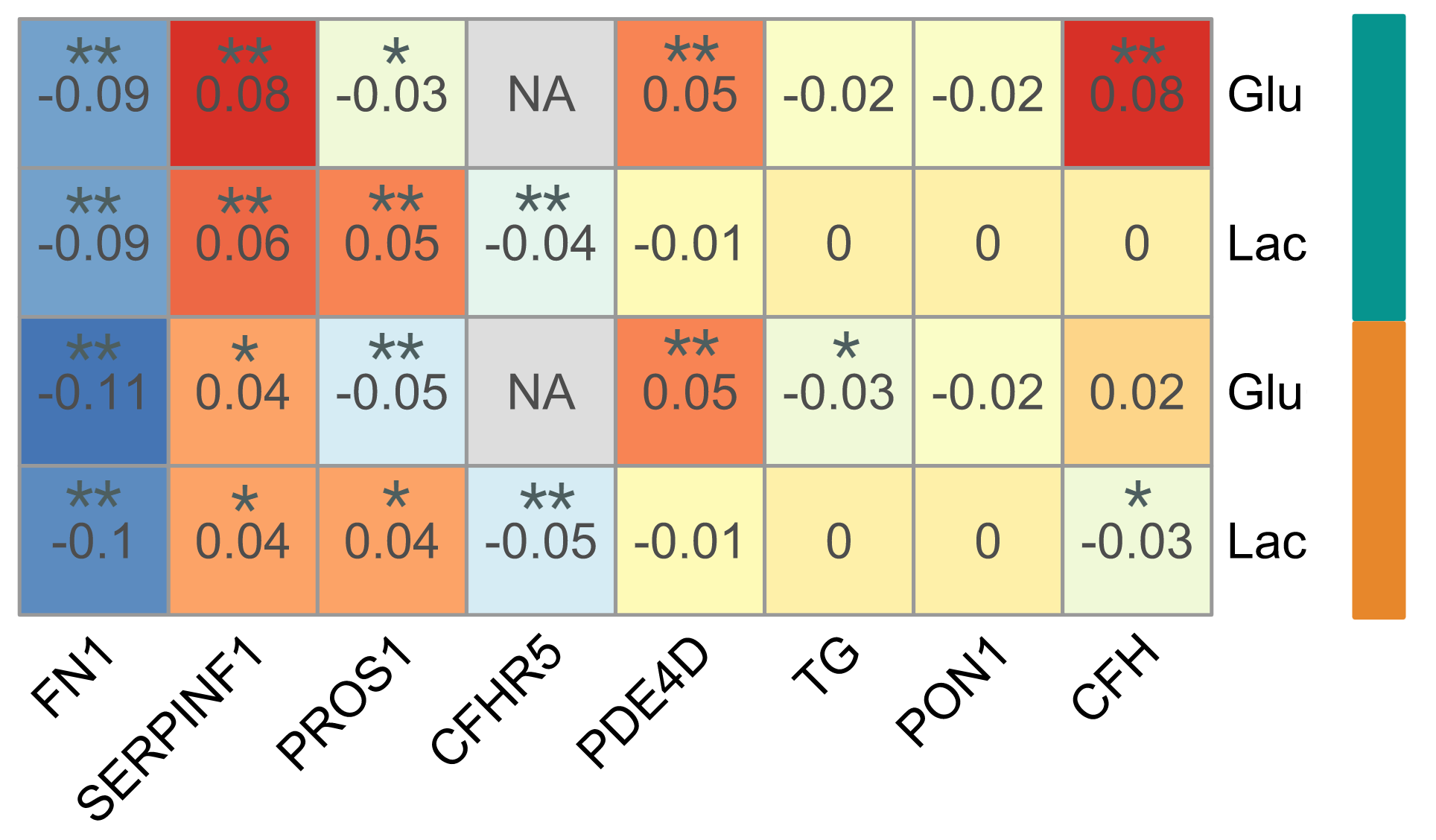

Supplement: Supplementary file 1 [file biology-13-00272-s001.zip › Figure S2.png]
